# On the Pathological Appearances Presented in Marsh Fever

**Published:** 1865-12

**Authors:** J. Forsyth Meigs

**Affiliations:** One of the Physicians to the Pennsylvania Hospital


### ON THE PATHOLOGICAL APPEARANCES PRESENTED IN MARSH FEVER.

By J. FORSYTH MEIGS, M.D., one of the Physicians to the Pennsylvania Hospital.

In the admirable work on diseases of the liver, by Dr. F. T. Frerichs, the first volume of which was published in Breslau, in 1858, and the second in 1861, and which has been translated for and published by the New Sydenham Society—a work which must take its place as a model of medical observation and research—will be found a chapter entitled, "The Pigment Liver; Melanæmic Liver; Alterations in the Liver resulting from Intermittent Fever." My attention having been attracted with unusual interest to the facts and doctrines portrayed in that part of the work, I became anxious to ascertain whether the same pathological appearances as those observed by Dr. Frerichs could be found in cases of malarial disease occurring in this country.

The following cases were observed in the wards of the Pennsylvania Hospital of this city, during the past autumn, and, though few in number, they are sufficient to attest the accuracy of the statements made by Dr. Frerichs:—

The first case was that of a young unmarried woman who, when brought to the hospital, had been suffering for six weeks with intermittent fever, contracted in one of the lower counties of Maryland. The disease was tertian at first, from which it became quotidian, and so continued until the patient entered the wards. No quinia had been exhibited, and the disease therefore presented its natural characters uninfluenced by specific treatment. The patient was not very much reduced in flesh, but was excessively feeble, blanched in color, and had lost all appetite. The spleen descended two inches below the margin of the chest. She had a chill every afternoon, followed by fever and sweating, but exhibited no other signs of disease. On pricking the finger and placing a drop of blood under a quarter-inch lens, it was seen that the red corpuscles were not more than one-fourth as numerous as in health, the white corpuscles were about normal in quantity, but what especially arrested attention was the presence in the field of numerous minute particles of irregular shape, with angular edges, of blackish color, and entirely opaque. These were the pigment granules of Dr. Frerichs.

The patient was put to bed. Sixteen grains of quinia were given daily, milk-punch, bread and milk, and beef-tea were ordered for diet, and the chills were arrested after the second day. Citrate of iron, five grains, three times a day, was ordered on the third day, the quinia was reduced to twelve, and then to six grains daily, and in a week the patient was convalescent.

Soon after this, three sailors were brought to the hospital from a vessel which had been lying four weeks in the harbor of Aspinwall, in the Isthmus of Panama, and which had been five weeks on the voyage from that port to Philadelphia.

I am indebted to two of the resident physicians of the hospital for notes of the cases, and for details of the *post mortem* examination, and of the microscopic appearances—to Dr. T. H. Andrews, for the former, and to Dr. Edw. Rhoads, for the latter.

Case I. Geo. H., aged 27, a native of New York, was admitted Nov. 12th, 1864. Whilst at Aspinwall, he had been seized with intermittent fever, at first tertian, and then quotidian in type. This was checked by quinia at first, then returned at one of the septenary periods, and continued until he reached the hospital. Owing to the disease and to poor fare on board ship, he had lost flesh rapidly. After admission, he had a chill every day, at one o'clock, lasting about half an hour, followed by fever and sweat. He had no appetite, suffered from constipation, was very pallid and feeble, but had no other material sign of disease, except decided enlargement of the spleen. A mild cathartic was exhibited; he was ordered thirty grains of sulphate of quinia in the first interval; milk-punch and beef-tea were given in quantities of a wineglassful every two hours alternately, and, the chills soon ceasing to occur, the quinia was reduced to sixteen grains. Under this treatment the patient improved rapidly and steadily, and, though still pale and weak, he decided to leave the hospital on the 21st.

Case II. G. S., aged 32, native of Ireland, admitted Nov. 12th, 1864. Has been a seaman for twenty years; was always strong and healthy, weighing about 150 lbs., but addicted to the use of alcohol to excess. About eight weeks before admission, whilst his ship was in the port of Aspinwall, was seized with intermittent fever, of a typhoid type, which was checked by the use of quinia. Soon after this, his legs became dropsical, so that when the vessel left Aspinwall he was disabled from duty. During the voyage to Philadelphia, the fare on board ship was very poor, so that when he reached the hospital he was greatly reduced in flesh and strength. He now had anasarca of the lower extremities and of the trunk; ascites; excessive tympany; copious, watery diarrhœa; pallid, cool, and moist skin; pinched and anxious countenance; pulse 88, and feeble; respiration 36, and labored. The tongue was coated, and the appetite not bad, but he could take only small quantities of food, owing to the gastric distress which it caused. The spleen was enlarged and tender on pressure. No abnormal sounds in the lungs; heart sounds healthy. The urine was normal in amount, alkaline, specific gravity 1013, highly albuminous, containing in the nebulous deposit many pigment flakes and granules of variable size and color, usually reddish-brown. Blood drawn from the finger exhibited under the microscope more than the normal proportion of white corpuscles, with several pigment granules in each field. The red corpuscles appeared gelatinous, and not only gave up their coloring matters in the added water, but, for the most part, entirely dissolved, or left only a filmy residue.

He was ordered sulphate of quinia, eight grains daily, compound spirits of juniper, two drachms four times a day, infusion of juniper, half a pint daily, and a diet of milk-punch and essence of beef, a wineglassful every two hours alternately. Laudanum enemata were occasionally given to check the diarrhœa. His condition remained much the same under this treatment, when his friends, in spite of his extreme illness and exhaustion, removed him from the hospital on the 24th of November.

Case III. W. L., sailor, aged 23, native of New York, was admitted November 12th, 1864. Five weeks before admission he had sailed from Aspinwall, after lying in that port during about four weeks. Whilst in port, and engaged in cleaning the paint on the ship's sides, he was overcome with heat and exposure, was seized with headache, rigors, pain in the back, and diarrhœa, so as to be forced to take to his bed. During the passage home, which lasted five weeks, he had poor and insufficient food, was very weak, unable to do duty, and, in addition, his malarial disease became scorbutic, so that he was emaciated, and had sores on the wrist, hip, sacrum, and back. When brought to the hospital, he was in a condition of profound cachexia. The pulse was frequent and feeble, the skin not very hot, the urine scanty, the feet œdematous, and he was excessively feeble. The heart and lungs presented no marked symptoms or signs of disease.

He was ordered quinia, four grains every four hours, tincture of chloride of iron, ten drops every four hours, milk-punch and beef-tea, of each a wineglassful every two hours alternately. He was also allowed spinach, onions, and lemonade. He had an opiate at bedtime. The bowels were moved by an enema when necessary. He improved slightly at first, then sunk again, and died November 18th.

*Autopsy* (*from notes by Dr. Rhoads,*) *fourteen hours after death.*—Figure of medium height; extreme emaciation and pallor; ill-conditioned ulcers over prominent osseous points; rigor mortis very slight; muscles atrophied; feet œdematous.

*Thorax.*—Pleura smooth, clear, and healthy throughout; one pint of limpid, straw-colored fluid surrounding each lung; about one ounce of similar effusion in the pericardium; heart flabby, slightly dilated, distended; in the left ventricle a soft, gelatinous, dark coagulum, with colored serum; in the right ventricle and auricle, extending through the tricuspid and pulmonary valves, intertwining with the cords and adhering to the walls, a firm, white, fibrinous clot. Lungs in posterior inferior portions hypostatically congested and imperfectly expanded, but everywhere inflateable.

*Abdomen.*—Peritoneum smooth and clear, containing nearly six ounces of serous fluid.

*Liver.*—Firm, of a dark bronze color; weight four and three-quarter pounds; section surface mottled by the marked congestion of hepatic veins; gall-bladder partially filled with bile.

*Spleen.*—About twice the natural size; weight twelve ounces; soft; almost black.

*Kidneys.*—Not enlarged materially; cones congested; conspicuous in section.

*Bowels.*—Pale; no ulceration or thickening of their mucous membrane. The epithelium in the colon disposed to separate as from the healthy membrane after maceration.

*Mesenteric glands.*—Healthy in appearance.

*Microscopic appearances.*—The splenic pulp presented its usual elements, with a large excess of blood. Corpuscles unchanged, or in various conditions of disintegration. Scattered in great abundance over the field, both free and within the nucleated corpuscles, pigment granules, irregular in shape and outline, rounded or angular, varying in color from absolute black, with the strongest light, to the lighter shades of reddish-brown at the edges or throughout, and in size from the 100th of a line in diameter to a mere point.

*Liver.*—Hepatic cells healthy. Pigment grains everywhere present; somewhat less numerous than in the spleen. They were not observed within the cell walls.

*Kidneys.*—Epithelial cells enlarged; incipiently fatty. More engorgement of the straight capillaries in the medullary than of those in the cortical portion. Similar pigment granules to those in the spleen and liver, but much less numerous.

*Blood in cardiac cavities.*—More than the normal proportion of white corpuscles. Marked deficiency of fibrin (except in old clot on right side). Red corpuscles individually much darker than in health; their coloring matter readily passing into and tinging water, on the addition of that fluid. Several pigment granules in each field.

*Heart.*—Muscular fibres healthy; capillaries filled with blood; an occasional aggregation of fine granules visible.

The *lungs* exhibited much pigment, but an excess over that usually found in the organs could not positively be affirmed.

*Remarks.*—Dr. Frerichs states that in individuals who die from the effects of marsh poison, there are frequently found peculiar changes in the liver, spleen, brain, kidneys, and blood, which evidently belong to the disease resulting from that poison. Believing that these discoveries have not been brought before the medical public of this country, as they deserve to be, I shall, in as few words as possible, quote them on this occasion.

The most important of these pathological appearances are the following:—The liver presents a steel-gray or blackish, or, not unfrequently, a chocolate color. This change in color is caused by pigment matter which is accumulated in the vascular apparatus of the gland. The larger part of the pigment is found in the capillary network of the portal and hepatic veins, but it is also found, in most cases, in the branches of the hepatic artery. The hepatic cells are said, by Frerichs, to remain exempt, he having in no case, observed any pigment in them, as has been asserted by Virchow. It is stated, however, in a note, that, after extravasations of blood into the hepatic parenchyma, deposits of red, brown, and black pigment in the cells are not unfrequently met with. In one case of cirrhosis he met with extensive masses of this nature. The cells were found either normal, or filled with brown bile; or sometimes infiltrated with oil, and occasionally, but only after a long continuance of the disease, they contained colloid or lardaceous matter.

In acute cases the size of the organ is either normal or enlarged; at later periods it diminishes in volume and undergoes a true atrophy, unless it have been infiltrated with colloid matter a condition which was met with only in rare instances.

The spleen undergoes similar changes. It is dark brown, or sometimes bluish-black, and its parenchyma contains large quantities of the same pigment as that found in the liver. In acute cases it is enlarged, softened, and congested. In less severe cases its volume is not much changed, unless, as seldom happens, it undergoes lardaceous degeneration, in which case its volume and consistence are considerably augmented.

The brain also exhibits the pigmentary deposits. The cortical substance assumes a chocolate or black-lead like hue, whilst the white matter remains unchanged, unless the amount of pigment be excessive, in which event the white matter presents a gray appearance, and its fine vessels resemble brown streaks. Under these circumstances microscopic examination shows the capillaries to be filled with black granules and scales.

The kidneys frequently participate in these changes. The cortical substance shows gray spots, and dark lines may be seen in the pyramids following the course of the bloodvessels. The microscope exhibits pigment matter in the capillaries of the cortical substance, and particularly in the Malpighian bodies, and sometimes isolated fragments are found in the uriniferous tubes.

The pigment is also found in the capillaries of the lungs. Dr. Frerichs says it is difficult to distinguish, in older persons, between the pigmentary deposits of another nature found in the lungs and those caused by malerial disease.

The pigment matter is found in abundance in the blood, and particularly in that of the portal vein, and is thence conveyed to the different tissues and organs in which it is discovered. The usual form is that of small, rounded, or angular granules, which are sometimes isolated, or more frequently connected together in groups by a pale substance, soluble in acetic acid and caustic alkalies. True pigment cells are observed along with the granules and granular masses, though in somewhat smaller quantity. They resemble in size and form the colorless corpuscles of the blood, or they consist of larger spindle or club-shaped cells and rounded nuclei, with sharply-defined walls. These cells contain a greater or smaller number of black granules. The color of the pigment is usually deep black, more rarely brown or orchre-colored, and, least frequently of all, reddish yellow.

It is thought by most observers that this pigment matter is formed in the spleen. Dr. Frerichs is of opinion that, though there are many reasons for such a belief, there is no proof that it may not be formed in other portions of the vascular system.

He believes, however, that there is no doubt the larger portion of the pigment is formed in the spleen.

In malarial disease, particularly, there is every reason to suppose that the spleen is the principal seat of formation of the pigment. During the congestions of that organ, which so constantly occur in this disease, the stagnation of the blood in the venous sinuses gives rise to changes which result in the formation of pigment in the masses of stagnant blood. Frerichs supposes that the club and spindle-shaped pigment cells are the epithelium of the lining membrane of the sinuses infiltrated with the decomposed red matter of the blood, that the globular pigment cells are colorless blood corpuscles infiltrated with molecules of coloring matter, and that the pigment scales are the broken-up fragments of the coagulum.

The results of these changes of the blood in the spleen are admirably drawn. One of the first effects is the production of chlorosis or anæmia, by the destruction of the red corpuscles, and no one who has seen the rapid advance of pallor in a case of unchecked or obstinate malarial disease can fail to be pleased with so admirable an exposition of the mode of production of the phenomenon. The pigment, carried by the spleen to the portal vein, and thence to the liver, is, in part, arrested in the capillaries of that organ, whilst other portions, consisting of the smaller granules and cells, pass on through the liver to the general circulation, and so reach the lungs, brain, and kidneys.

It is this retention of the pigment matter in the capillaries of the different organs by which Dr. Frerichs explains many of the symptoms which accompany certain of the severer forms of the disease. Thus, the retention of the coarser fragments in the capillaries of the liver, by clogging those vessels, gives rise to stasis of blood in the venous radicals of the portal vein. This stasis explains the intestinal hemorrhages and the diarrhœas which so frequently attend the more violent cases of bilious fever. In other instances, in addition to the effusions taking place from the mucous membrane of the alimentary canal, we have serous effusion into the peritoneal sac, occasioning ascites, a result similar in cause and symptoms to the ascites caused by cirrhosis.

The cerebral phenomena, the stupor, delirium, convulsions, or paralysis, which sometimes occur in miasmatic fever cannot, according to our author, be so satisfactorily shown to depend on the retention of pigment in the capillaries of the brain, as the intestinal hemorrhages, diarrhœa, and ascites, have been shown to be the result of hipatic obstruction, but that a connection between the two does really exist, at least in some degree, can scarcely be doubted.

In many severe cases, it was found that the albuminuria and general dropsy present during life, evidently resulted from the obstruction to the renal circulation occasioned by the retention of pigment in the capillaries of the kidneys, and particularly in the Malpighian bodies.

Frerichs states that, considering the great frequency of intermittent fevers, the cases in which there is a marked development of pigment are comparatively rare; hence, in such cases other agencies, of which we possess no accurate information, must co-operate with the usual causes of intermittent fever. In the present defective state of our knowledge as to the nature of infectious diseases, it cannot be determined whether a particular quality, or an unusual intensity of miasm, is necessary for the purpose."—The cases which he has observed and published occurred after an inundation in Selesia, resulting from an overflow of the Oder, in 1854. Since that period cases of this kind have been very seldom observed, although the ordinary forms of intermittent fever are never absent. He remarks that a perfectly acurate diagnosis can only be made by direct examination of the blood. A few drops carefully collected are sufficient to show the presence or absence of large quantities of pigment. He usually collects it by means of a cupping-glass, care being taken to prevent the admixture of foreign matter.

The four cases given above are all well-marked examples of the disease, the pathological results of which Dr. Frerichs describes so well. The first case is one of simple intermittent, arising in one of the higher latitudes subject to marsh miasm, a form easily overcome by proper treatment in the early stage, and yet, even in this case, owing to its being allowed to run on unchecked for some weeks, we found in the blood of the patient, taken from the finger, the peculiar pigment granules described by Frerichs.

The other three cases were of a very different type. Originating in one of the lower latitudes, they exhibit a severity which gives us the opportunity of observing the serious consequences resulting from a dose of marsh miasm of the most poisonous kind. One of the three, though very ill when he reached the hospital, was saved by proper treatment. One was removed from the hospital in a condition which precluded all reasonable hope of his recovery. The third died. This case presented all the severest effects which result from the action of the malarial poison on the human economy; periodical fever, rapid loss of strength and flesh, with sudden production of anæmia, and, somewhat later, diarrhœa, ascites, anasarca, in connection with albuminous urine, general cachexia, and death. With Dr. Frerichs's work before us it is easy to understand the successive steps of these changes. The congestion and stagnation of blood in the spleen explain, by the actual destruction of the red globules, the speedy induction of anæmia. This condition is also greatly favored, in all probability, by the failure of the globule-making function of the gland. As the destruction of the globules proceeds, their red matter is converted into pigment, which pigment, in its various forms, is carried into the vascular apparatus of the body. Deposited in the liver it may accumulate to such an extent finally as to impede the portal circulation and give rise to diarrhœa and ascites. Lodged in the vessels of the kidneys, and especially in the Malpighian bodies, it may cause a like impediment in those organs, and there is developed a true albuminuria with its consequent anasarca. In the third case given above we had all these conditions. Pigment in the liver, with diarrhœa and ascites. Pigment in the kidneys, with albuminous urine and anasarca.

The lessons as to treatment to be drawn from these discoveries are simple. The beneficent action of cinchona can scarcely be raised higher than before, but its precise mode of action is more clearly developed. Its antiperiodic power is that which makes it invaluable. By cutting off the paroxysms it prevents the repeated congestions and stagnations of blood in the spleen, and thus arrests the destruction of the blood in that organ, and the consequent production of pigment, which is the obstructive agent carried into the more distant organs of the body. The one most valuable therapeutic law taught by these facts is the necessity of applying the saving agent early in the case. When so used it averts the disastrous effects of repeated paroxysms, and thus saves life as clearly and distinctly as does a surgical operation in a case where no other course of procedure is possible. Reason deduces this result as plainly in the former case as the eye beholds it in the latter.—*Am. Jour. Med. Sciences.*

---

A NEW medical school is announced at Cairo, Ill., by Dr. Jos. N. M'Dowell, late of the Missouri Medical College, and Dr. C. W. Dunning, both of whom will be professors in the new school.—*Boston Med. & Surg. Jour.*

